# Cthrc1, a Novel Circulating Hormone Regulating Metabolism

**DOI:** 10.1371/journal.pone.0047142

**Published:** 2012-10-08

**Authors:** J. Patrizia Stohn, Nicole G. Perreault, Qiaozeng Wang, Lucy Liaw, Volkhard Lindner

**Affiliations:** Center for Molecular Medicine, Maine Medical Center Research Institute, Scarborough, Maine, United States of America; University of Cordoba, Spain

## Abstract

**Background:**

We discovered the gene Collagen Triple Helix Repeat Containing 1 (Cthrc1) and reported its developmental expression and induction in adventitial cells of injured arteries and dermal cells of skin wounds. The role of Cthrc1 in normal adult tissues has not yet been determined.

**Methodology/Principal Findings:**

We generated mutant mice with a novel Cthrc1 null allele by homologues recombination. Cthrc1 null mice appeared developmentally normal. On the C57BL/6J background, livers from Cthrc1 null mice accumulated vast quantities of lipid, leading to extensive macrovesicular steatosis. Glycogen levels in skeletal muscle and liver of Cthrc1 null mice on the 129S6/SvEv background were significantly increased. However, Cthrc1 expression is not detectable in these tissues in wild-type mice, suggesting that the lipid and glycogen storage phenotype may be a secondary effect due to loss of Cthrc1 production at a distant site. To investigate potential hormonal functions of Cthrc1, tissues from adult mice and pigs were examined for Cthrc1 expression by immunohistochemistry with monoclonal anti-Cthrc1 antibodies. In pigs, Cthrc1 was detected around chromophobe cells of the anterior pituitary, and storage of Cthrc1 was observed in colloid-filled follicles and the pituitary cleft. Pituitary follicles have been observed in numerous vertebrates including humans but none of the known pituitary hormones have hitherto been detected in them. In C57BL/6J mice, however, Cthrc1 was predominantly expressed in the paraventricular and supraoptic nucleus of the hypothalamus but not in the posterior pituitary. In human plasma, we detected Cthrc1 in pg/ml quantities and studies with ^125^I-labeled Cthrc1 revealed a half-life of 2.5 hours in circulation. The highest level of Cthrc1 binding was observed in the liver.

**Conclusions:**

Cthrc1 has characteristics of a circulating hormone generated from the anterior pituitary, hypothalamus and bone. Hormonal functions of Cthrc1 include regulation of lipid storage and cellular glycogen levels with potentially broad implications for cell metabolism and physiology.

## Introduction

We originally discovered collagen triple helix repeat containing 1 (Cthrc1) in a screen for novel sequences induced in rat carotid arteries upon balloon catheter injury [1]. The response to this injury results in constrictive remodeling with reduction in lumen size and fibrosis of the adventitia. Cthrc1 was not expressed in normal vessels, but was induced in adventitial cells in remodeling arteries. In addition, Cthrc1 expression was observed in dermal fibroblasts during skin wound healing [1].

Targeted replacement of the first exon of the Cthrc1 gene by a LacZ reporter gene in mice was reported to demonstrate expression of Cthrc1 in inner ear hair cells [2]. This study described abnormalities in inner ear development when Cthrc1 null mice were crossed with mice carrying one mutant allele of Vangl2, but these abnormalities were only observed when the compound mutants were on a mixed 129/SvEv-C57BL/6 genetic background and not when the mutants were crossed with outbred CD-1 mice. In connection with in vitro data derived from co-cultures of transfected HEK293T, the authors concluded that Cthrc1 is involved in non-canonical Wnt signaling as part of the planar cell polarity pathway [2]. A separate mutant Cthrc1 mouse with deletion of exon 2 was reported to have reduced bone mass [3].

Expression analyses at the RNA level using in situ hybridization have identified the sites of Cthrc1 expression during embryonic development. In addition, our studies have also shown that Cthrc1 is expressed by the activated fibroblast of remodeling tissues following injury [4]. Whether Cthrc1 protein is constitutively expressed in any tissues of normal adult animals has so far remained unclear largely because reliable antibodies suitable for detection of Cthrc1 at the cellular level were not available.

The pituitary gland is the master endocrine gland, with the anterior pituitary expressing and secreting a variety of hormones and the posterior pituitary releasing oxytocin as well as vasopressin expressed by neurosecretory cells of the hypothalamus. Colloid-filled follicles of the anterior pituitary containing PAS (periodic-acid Schiff reaction) positive material have been reported in several vertebrates including humans [5], [6]. These follicles have been known to increase in number and size with age. The content of the follicles was reported to include polysaccharides and glycoproteins but none of the known pituitary hormones have hitherto been localized to them. To our knowledge, the function and significance of these follicles is still unknown.

Here we generated mutant mice with a novel targeted Cthrc1 null allele and focused on the analysis of their phenotype in adulthood. Monoclonal antibodies were generated against C terminal and N terminal epitopes of Cthrc1, which allowed us to localize Cthrc1 in tissues of a variety of species including pig. Our results demonstrate circulating levels of Cthrc1 in human plasma including expression in the anterior pituitary as well as neurosecretory nuclei of the hypothalamus.

## Materials and Methods

All protocols involving animals were approved by the Institutional Animal Care and Use Committee of the Maine Medical Center (protocol numbers 0905 and 1112) and were in compliance with all applicable regulations and guidelines including the National Institutes of Health *Guide for Care and Use of Laboratory Animals*. All surgical interventions were performed under general anesthesia with tribromoethanol/tert. amyl alcohol. Human plasma samples were obtained under a protocol approved by the Institutional Review Board of the Maine Medical Center (protocol number 3657).

### Animals

**Figure 1:**
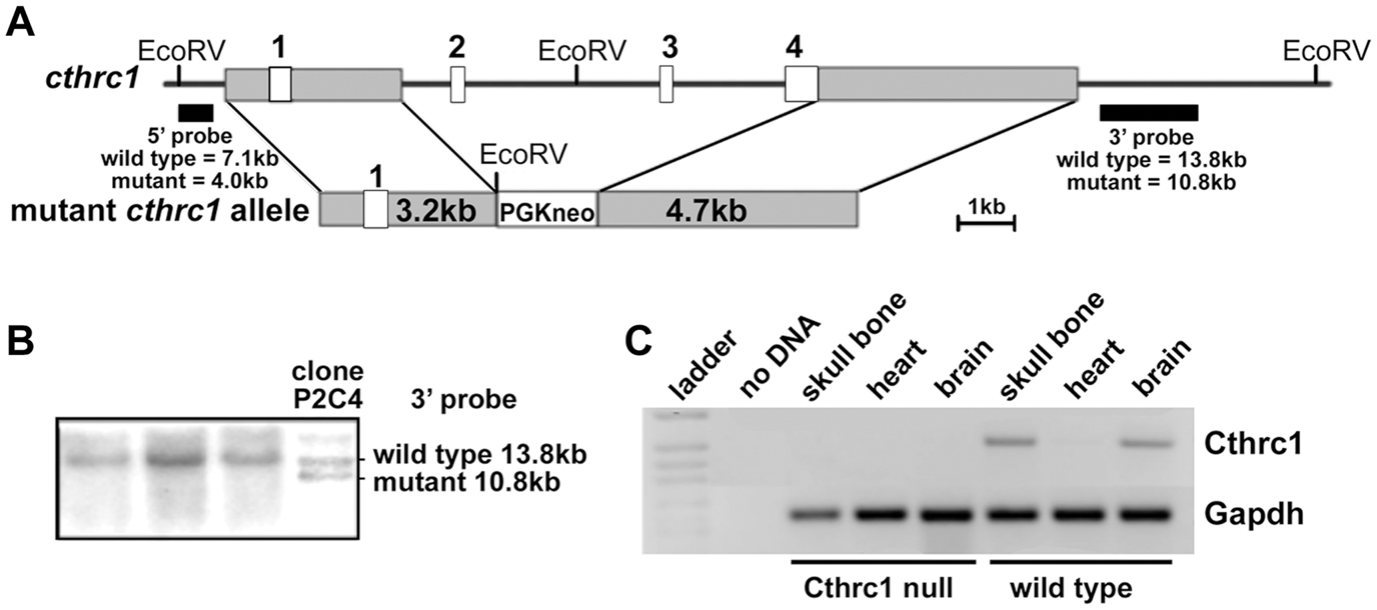
Generation of Cthrc1 null mice. (A) Cthrc1 gene targeting schematic showing replacement of exons 2–4 with a PGK-neo cassette. (B) Embryonic stem cell targeting with Southern blot analysis of genomic DNA from ES cell clones shows a targeted clone. (C) RT-PCR shows lack of Cthrc1 transcripts in RNA preparations of skull bone and whole brain of adult Cthrc1 mutants.

We generated a novel Cthrc1 null allele by replacing exons 2, 3 and 4 with a neomycin cassette using the targeting vector pKO Scrambler NTKV-1905 (Stratagene) (Fig. 1A). Exon 1 contains only 5′ untranslated sequence and the N-terminal 52 amino acids, which include the signal sequence plus additional 20 amino acids. Embryonic stem cell clones were screened by Southern blotting and positive clones injected into blastocysts (Fig. 1B). Two chimeras were obtained and bred with C57BL/6J mice (Jackson Laboratory), producing offspring with agouti coat color indicating germ line transmission. Their genotypes were verified by Southern blotting and a PCR screen that amplified the wild-type allele (primers: 5′-CCACTGGAAACCTCTGGAGTTG-3′ and 5′-AAGTTCACACAAAGGAAGCCCCGC-3′) and the mutant allele (primers: 5′-GTGTGTTTTGAGGTGTGGTCCC-3′ and 5′TGGATGTGGAATGTGTGCGAGG-3). These animals have been backcrossed on the 129S6/SvEv (Taconic) or C57BL/6J background for 11 generations. Cthrc1 null mice for experiments were derived from matings of homozygous mutants. The mutant allele was backcrossed regularly with 129S6/SvEv or C57BL/6J breeder stock to prevent genetic drift. The age of the mice used for specific experiments is indicated in the figure legends.

Mice were fed a standard rodent diet (Harlan, 2018 Teklad Global 18% Protein Rodent Diet) and water ad libitum, and housed with dry cellulose bedding (Harlan, 7070 Diamond) under a 14 hour daylight-10 hour night cycle. Litter size was determined as the number of pups weaned at the age of 21 days after birth. Growth curves were obtained for Cthrc1 null and wild type mice on the 129S6/SvEv (n = 5–33 per time point).

Livers of seven month old mixed gender Cthrc1 null and wild type mice on the C57Bl/6J background were examined to assess histology and lipid content. Lipid storage was determined by image analysis of oil red O stained sections using the same approach as described previously [4].

Pig tissues were obtained from euthanized animals (6 to 12 months of age) of the Advanced Trauma Operative Management (ATOM) courses conducted at our facility or from a local slaughterhouse. Primer sequences used for RT-PCR of pig cDNA were 5′- GACCCCTTCATTGACCTCCACTAC-3′ and 5′-ACATACTCAGCACCAGCATCGC-3′ for Gapdh and 5′-GAATGCCTGAGGGAAATCTTTGAG-3′ and 5′-CGTCTCCTTTTGGGTAATCTGCG-3′ for Cthrc1. The annealing temperature was 55.9°C and 35 cycles of amplification were performed.

Rat tissues from three month old male Sprague Dawley rats were kindly provided by Dr. Renée LeClair (University of New England, Biddeford, ME).

### Glycogen Assay

Mixed gender wild type and Cthrc1 null mice on the 129S6/SvEv background were used for this assay (n = 4–23 animals per group, 3–5 months of age). Samples were obtained from identical sites of the liver and the right gastrocnemius muscle. Glycogen from 10 mg fresh-frozen tissue was hydrolyzed to glucose in 500 µl of 2 N HCl for 2 h at 100°C with vortexing every 30 minutes. Samples were neutralized with 500 µl 2 N NaOH and 50 µl 1 M TRIS pH = 7.6. The glucose hexokinase reagent (ThermoFisher) was used as directed. A standard curve was established with glucose standards covering the range of 0.00312 to 0.1 µmole. Using GraphPad Prism software, concentrations of glucose in the samples were calculated based on the standard curve. Three different samples from each organ were assayed in duplicates and mean values were calculated and compared using Student’s t-test. The assay was performed four times with separate sets of samples from different mice and similar results were obtained each time. The results of a representative experiment are shown.

### Body Fat and Blood Triglyceride Levels

Body composition was determined as described using a Lunar PIXImus II Mouse Densitometer (GE Medical Systems) [7]. Blood triglyceride levels were determined in non-fasted 3–4 month old mixed gender wild type and mutant mice on the 129S6/SvEv background using test strips for the One Touch Ultra system.

### Glucose Tolerance and Insulin Stress Test

For glucose tolerance testing, mixed gender wild type and Cthrc1 null mice on the 129S6/SvEv background (n = 8, 3–4 months of age) were injected intraperitoneally with 1 g of glucose per kg of body weight after 16 hours of fasting. For insulin stress testing, mixed gender, non-fasted wild type and Cthrc1 null mice on the 129S6/SvEv background (n = 8, 3–4 months of age) were injected intraperitoneally with insulin (1 U/kg, NovoLog, Novo Nordisk). Blood was obtained for glucose level measurements using the One Touch Ultra glucometer at the indicated times.

### Rabbit Monoclonal Antibody Generation, Immunohistochemistry, and Immunoblotting

**Figure 2:**
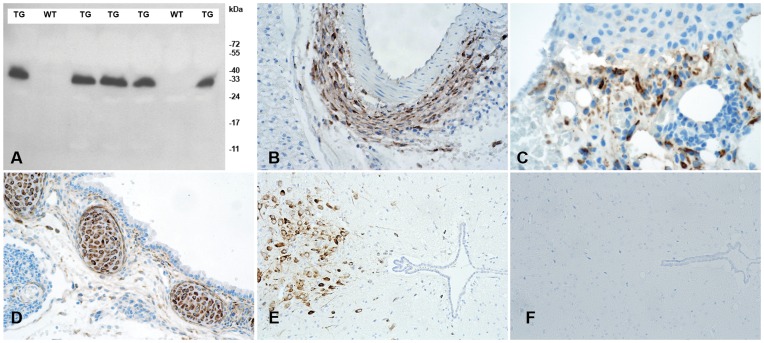
Characterization of rabbit monoclonal anti-Cthrc1 (Vli-55) antibody. (A) Western blot analysis of plasma from Cthrc1 transgenic mice (TG) and their negative wild type littermates (WT). (B) Cthrc1 immunohistochemistry shows expression in adventitial cells of the remodeling renal artery following angiotensin II infusion, (C) dermal cells in 7 day old skin wounds, and (D) embryonic cartilage (E17.5). (E) Cthrc1 expression is restricted to the midbrain in wild type 129S6/SvEv mice and (F) no expression is detected in the midbrain of a corresponding Cthrc1 null mouse.

A rabbit monoclonal antibody was raised against a synthetic peptide of the conserved C terminus of Cthrc1 (GDASTGWNSVSRIIIEELP) using the services of Epitomics, Inc. (Burlingame, CA). Clone Vli-55 was suitable for Western blotting and immunohistochemistry on paraffin-embedded, paraformaldehyde-fixed tissues. Rabbit monoclonal Vli-55 was used at 20 ng/ml for immunohistochemistry on paraffin-embedded, formalin-fixed tissue section following antigen retrieval with citrate buffer (0.1 M, pH = 6.0). Subsequent steps of the immunostaining procedure were executed as previously published [7]. We conjugated the antibody to horse radish peroxidase (Pierce, ThermoFisher) following the manufacturer’s protocol and validated its suitability for Western blotting using plasma samples from Cthrc1 transgenic and wild-type mice (1∶2000 dilution as described [1]. Five µl of plasma were loaded per lane and immunoblotting was performed on reduced and denatured samples (Fig. 2). Validation for immunohistochemistry was performed on tissues previously shown to express Cthrc1, i.e. adventitial cells of remodeling arteries, dermal cells in skin seven days after wounding, embryonic cartilage, and absence of staining on tissue sections from Cthrc1 null mice (Fig. 2). Pre-absorption of the antibody with peptide antigen was used as an additional control for specificity.

### Cthrc1 in Human Plasma

For detection of human Cthrc1 in plasma, mouse monoclonal antibodies were raised against a synthetic peptide with sequence of the N terminus of human Cthrc1 (SEIPKGKQKAQLRQRE) using the hybridoma services of Maine Biotechnology Services (Portland, ME). Anti-Cthrc1 clones 10G7 and 19C7 detected human Cthrc1 expressed in CHO-K1 cells by indirect ELISA without amplification in the low picogram range. Protein A-purified antibodies were conjugated to magnetic beads (Pierce, ThermoFisher) following the manufacturer’s protocol. EDTA plasma was obtained from healthy volunteers. 15 ml of plasma were incubated overnight at 1°C with anti-Cthrc1 conjugated magnetic beads, and washed extensively with phosphate buffer prior to elution with 0.1 M glycine, pH = 2.6. The eluate was immunoblotted with HRP-conjugated monoclonal anti-Cthrc1 antibodies following SDS-PAGE under reducing conditions.

### Labeling of Cthrc1 Protein with ^125^I(odine)

An adenovirus was generated expressing rat Cthrc1 with a C terminal myc/6×His tag. CHO-K1 cells were transduced with this adenovirus and Cthrc1 protein was purified from the conditioned medium with HIS-Select affinity gel (Sigma) following the supplier’s instructions. Silver-stained SDS-PAGE gels demonstrated >95% purity of the purified protein (not shown). A BCA protein assay (Pierce) was used to determine the concentration of the purified protein. Purified Cthrc1 was labeled with ^125^I (Perkin Elmer) using iodination tubes (Pierce). Six µg of radioactive labeled Cthrc1 were infused into adult anesthetized Cthrc1 null mice via the left carotid artery (n = 3 mice). Blood samples were obtained at indicated times and Cthrc1 levels were determined in a gamma counter. The half-life in circulation was calculated from the clearance curve. SDS-PAGE analysis followed by autoradiography was performed on 1 µl of plasma obtained thirty minutes after injection of ^125^I-Cthrc1 to verify its integrity. All tissues were harvested six hours after ^125^I-Cthrc1 injection following extensive perfusion with lactated Ringer’s solution to remove as much blood from organs as possible. ^125^I-Cthrc1 per mg wet weight of tissue was measured by gamma counting.

### Cell Culture and Western Blotting

HEK293-T and CHO-K1 cells were grown as described and transfected with an expression vector for human Cthrc1 using Fugene6 HD (Roche) [4]. 48 hours after transfection the growth medium was replaced with serum-free medium and cell lysates as well as conditioned media were harvested 24 hours later for immunoblotting with HRP conjugated anti-Cthrc1 antibody.

### Statistical Analysis

Data are expressed as means ± standard deviation. Student’s t-test was used for all calculations. P≤0.05 was considered significant.

## Results

### Generation and Characterization of the Cthrc1 Null Allele

To characterize Cthrc1 function *in vivo*, we generated a novel Cthrc1 null allele by replacing three of the four exons (exons 2–4) with a neomycin cassette (*Cthrc1^tm1Vli^*) (Fig. 1A). This mutant allele results in mice with no detectable Cthrc1 transcript in organs where it is normally expressed, such as bone and brain (Fig. 1C). Mice were backcrossed more than eleven generations to obtain the mutant allele on a 129S6/SvEv or C57BL/6J genetic background. Cthrc1 null mice were viable and displayed no obvious developmental abnormalities.

To verify that our Cthrc1 allele is a null mutation, we generated and characterized anti-Cthrc1 antibodies. We succeeded in developing highly sensitive and specific rabbit monoclonal antibodies against the conserved C terminus of Cthrc1. These antibodies allowed us to detect Cthrc1 by immunoblotting and in formalin fixed, paraffin embedded tissue sections by immunohistochemistry (Fig. 2).

**Figure 3:**
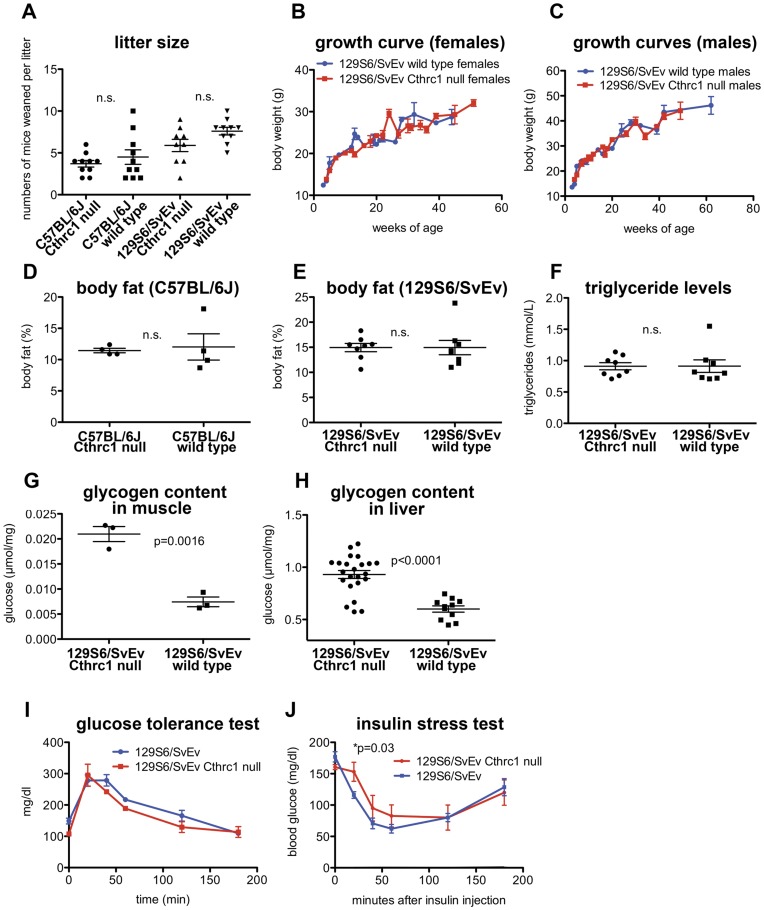
Normal growth but increased glycogen storage in liver and skeletal muscle of 129S6/SvEv Cthrc1 null mice. (A) Differences in litter size were not significantly different (n.s.). (B) Growth curves for females and (C) males were similar (n = 5–33 mice per time point). (D, E) Total body fat expressed as a percentage of body weight was similar in 8 week old female mice among mutants and wild type mice. (F) Blood triglyceride levels in 3–4 month old male and female mice were similar. (G) Glycogen storage in skeletal muscle and (H) liver is significantly increased in Cthrc1 null mice on the 129S6/SvEv background (males and females, 3–5 months of age). (I) Glucose handling is normal in Cthrc1 null mice (n = 8 per group, mixed gender, 3–4 months old). (J) Reduced insulin sensitivity was observed in Cthrc1 null mice (n = 8 per group, mixed gender, 3–4 months old).

The average litter size was slightly lower for Cthrc1 null mice, however, this did not reach a statistically significant level (Fig. 3A). Growth curves were established for Cthrc1 null mice on the 129S6/SvEv background and they were similar for Cthrc1 null mice and wild type mice (Fig. 3B, C). The percentage of fat of total body weight was measured in eight week old female mice by dual energy x-ray absorptiometry, and this analysis detected no differences between Cthrc1 null and wild type mice (Fig. 3D, E). In addition, blood triglyceride levels were also similar comparing Cthrc1 null and wild type mice (Fig. 3F).

### Metabolic Alterations in Adult Cthrc1 Null Mice

Liver histology with staining of sections for carbohydrates with the Periodic Acid Schiff (PAS) stain suggested increased carbohydrate storage in hepatocytes of Cthrc1 null mice on the 129S6/SvEv background. Quantification of the glycogen content in liver tissue with biochemical methods confirmed increased glycogen content in livers from Cthrc1 null mice (Fig. 3G). Measurements of glycogen content in skeletal muscle also consistently showed elevated levels of glycogen in Cthrc1 null mice compared to wild types (Fig. 3H). A glucose tolerance test revealed normal glucose handling with similar fasting blood glucose levels in Cthrc1 null and wild type mice (Fig. 3I). However, insulin sensitivity was slightly reduced in the mutants with significantly higher blood glucose levels at 20 minutes after insulin administration (Fig. 3J).

**Figure 4:**
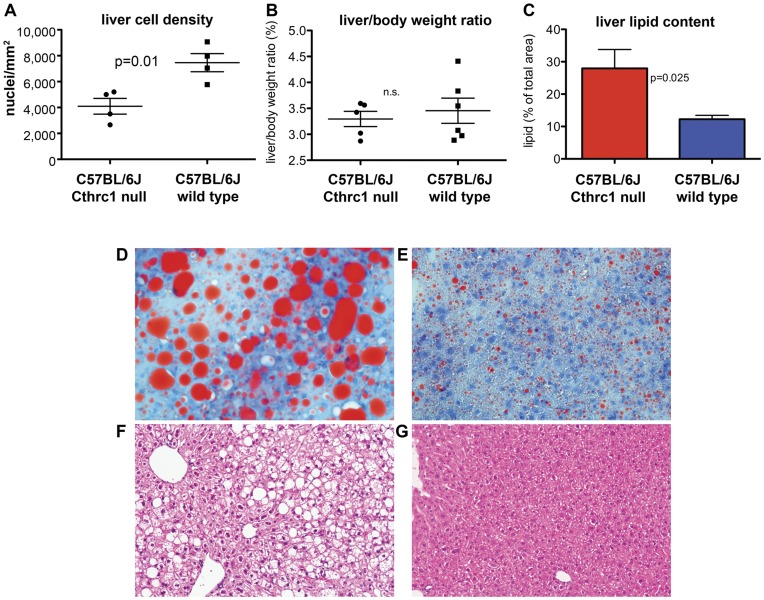
Fatty liver phenotype in C57BL/6J Cthrc1 null mice. (A) Cell density in livers of wild type and Cthrc1 null mice (n = 4 per group). (B) Liver to body weight ratio was similar among groups. (C) Lipid content in liver was determined by image analysis on oil red O stained sectins of 7 month old mixed gender Cthrc1 null and wild type mice (n = 6 per group). (D) Oil red O stain of a liver section from a Cthrc1 null mouse shows macrovesicular and microvesicular steatosis and (E) only minor lipid accumulations in a wild type mouse. (F) Hematoxylin & eosin stain of a liver section from a Cthrc1 null mouse and (G) a wild type mouse.

**Figure 5:**
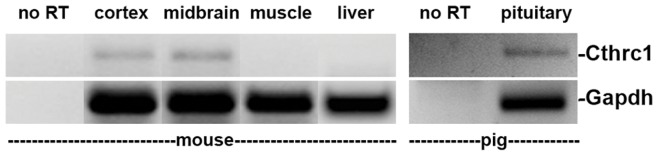
Cthrc1 expression in the pig pituitary but absence in adult mouse liver and skeletal muscle. RT-PCR with Cthrc1 specific primer pair was performed on the indicated mouse and pig RNA samples.

Routine histology of livers from seven month old Cthrc1 null mice on the C57BL/6J background revealed strikingly larger hepatocytes compared to wild type controls. Overall this resulted in a significantly lower cell density (Fig. 4A). The liver to body weight ratio was similar for Cthrc1 null mutants and wild type mice (Fig. 4B). We performed oil red O staining to determine whether lipid content was increased in livers from mutants and this revealed increased lipid storage in Cthrc1 null mice resulting in macro- and micro-vesicular steatosis (Fig. 4C–G). The abnormalities observed in liver and skeletal muscle of Cthrc1 null mice raised the question whether Cthrc1 was expressed in these organs. We were unable to detect Cthrc1 in normal adult livers and skeletal muscle at the protein level (data not shown) or by RT-PCR (Fig. 5). This led to the consideration whether Cthrc1 may be a circulating factor acting as a hormone.

### Expression of Cthrc1 in the Central Nervous System and Pituitary Gland

**Figure 6:**
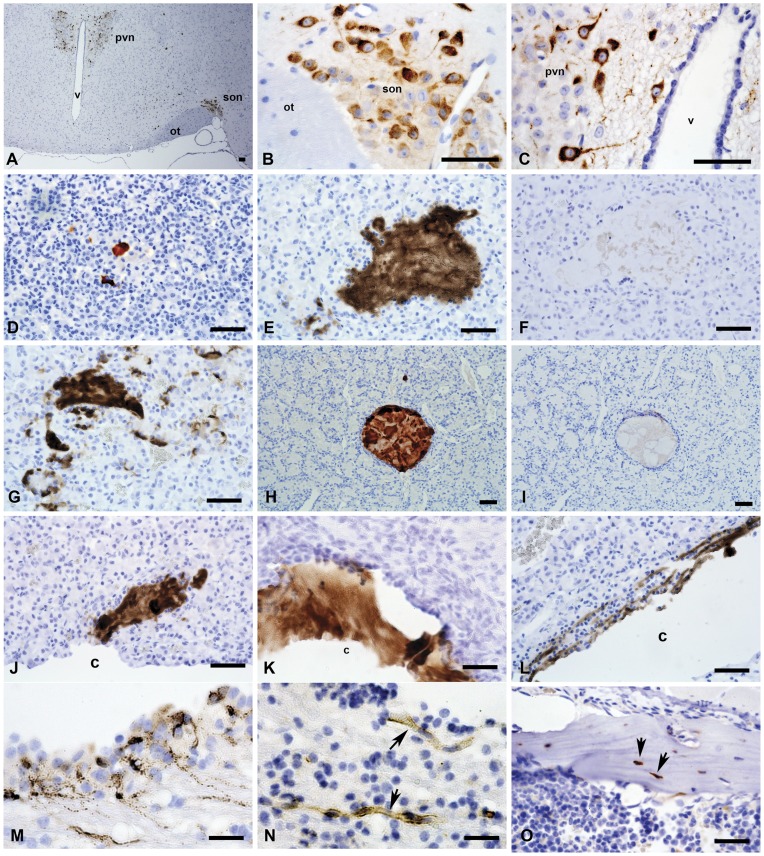
Cthrc1 immunohistochemistry of the brain and pituitary gland from mouse and pig. Sections of mouse (A–D) and pig (E–N) brain and pituitary were immunostained for Cthrc1 expression: (A) Low power view of a coronal section of the hypothalamus from a C57BL/6J mouse showing Cthrc1 immunoreactive cells in the paraventricular nucleus (pvn) and the supraoptic nucleus (son), ot = optic tract, v = third ventricle. (B) High power view of supra optic nucleus and (C) paraventricular nucleus. (D) Cthrc1 positive follicle in the anterior lobe of a 7 month old C57BL6/J male, (E) extensive accumulations of Cthrc1 in the anterior lobe of a pig pituitary, and (F) pre-absorption of antibody completely eliminates staining on an adjacent section. (G) Cytoplasmic localization of Cthrc1 in cells of the anterior lobe indicates expression. (H–I) are serial sections of a typical colloid-filled follicle of the anterior pituitary with (H) showing extensive immunoreactivity, (I) which is completely eliminated by pre-absorbing the antibody with peptide antigen (H). Also note the encapsulation of the follicle by folliculostellate cells. (J, K) Cthrc1 localization in the pituitary cleft (c) and (L) canaliculi connecting to the cleft. (M) An isolated area of cells in the paraventricular zone of the lateral ventricle expresses Cthrc1 (note granular appearance), and (N) nearby small vessels (arrows) contain Cthrc1. (O) Cthrc1 is expressed by some osteocytes (arrows) and osteoblasts in adult mouse bone. Scale bar = 50 µm.

We undertook a comprehensive immunoblot-based and immunohistochemistry-based survey of Cthrc1 expression in tissues from adult mice, rats and pigs. Expression of Cthrc1 in two month old C57BL/6J mice was detected in the supraoptic nucleus (son, Fig. 6A, B) and paraventricular nucleus (pvn, Fig. 6A, C) of the hypothalamus, both of which contain neurosecretory cells with axon terminals in the posterior pituitary. Serial sectioning of the entire midbrain region, however, did not indicate that any of Cthrc1 immunoreactivity of the hypothalamus was transported to the posterior pituitary for secretion (data not shown). We did not detect Cthrc1 expression anywhere in the pituitary of mice at this age nor did we find colloid-filled follicles in the anterior pituitary in mice at two months of age. Similarly, neurons of the supraoptic nucleus in pigs also expressed Cthrc1 but no Cthrc1 was found in the posterior pituitary (data not shown).

In wild-type mice older than six months of age, we occasionally observed small accumulations of Cthrc1 immunoreactivity in the anterior pituitary (Fig. 6D). In all pig pituitaries that we obtained, Cthrc1 immunoreactivity was variable in extent, but was consistently localized in the anterior lobe (Fig. 6E, G). Some of the Cthrc1 was found inside the cytoplasm of cells suggesting synthesis by those cells (Fig. 6G). Often Cthrc1 was observed in the lumen of colloid-filled follicles that were lined by folliculostellate cells (Fig. 6H–I). Preincubation of the antibody with the peptide antigen completely abolished the immunoreactivity, demonstrating specificity of the antibody (Fig. 6F, I). Colloid-filled follicles of the anterior pituitary, increasing in number and size with age, have been reported in numerous vertebrates including humans [5], [6]. Colloid material has also been reported in the pituitary cleft with adenohypophysial canaliculi connecting to it [8]. Cthrc1 accumulations were found close to the cleft (Fig. 6J) and also inside the pituitary cleft (Fig. 6K), often with small canaliculi carrying Cthrc1 connecting to the cleft (Fig. 6L). The significance of pituitary colloid follicles has yet to be determined, as none of the known pituitary hormones have been localized to them. Here we demonstrate that these follicles store Cthrc1.

As shown above, certain areas of the brain constitutively express Cthrc1 but we currently have no evidence that Cthrc1 from those sites enters the circulation. In brains from pigs, we found foci of paraventricular cells of the lateral ventricles that expressed Cthrc1 (Fig. 6M) and these sites are known to harbor neuronal stem cells. Close to the Cthrc1 expressing paraventricular cells we observed Cthrc1 immunoreactivity inside the lumen of small vessels (Fig. 6N), which provides indirect evidence that Cthrc1 may be secreted into the circulation. In young animals many tissues are still growing and undergoing remodeling, including the skeletal system, which constantly remodels. We did indeed find Cthrc1 expression in some osteocytes and osteoblasts (Fig. 6O) raising the possibility that Cthrc1 derived from bone may contribute to hormonal effects on liver and muscle.

**Figure 7:**
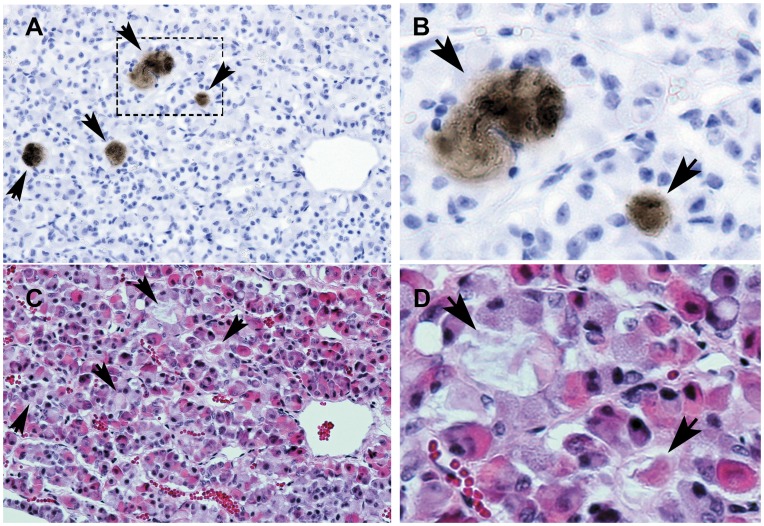
Cthrc1 accumulation in areas surrounded by chromophobe cells of the anterior pig pituitary. Adjacent sections were stained for Cthrc1 (A) and morphology (C, H&E). (B) and (D) are enlarged images of the boxed area shown in (A). Arrows indicate areas of Cthrc1 accumulation. Scale bar = 50 µm.

In the anterior pituitary immunoreactive Cthrc1 was observed in close association with chromophobe cells (Fig. 7A–D) suggesting that these cells express Cthrc1. Until now, no specific hormone has been reported to be expressed by chromophobe cells of the anterior pituitary lobe. Expression of Cthrc1 in the pituitary gland of pigs was confirmed at the mRNA level by RT-PCR (Fig. 5).

### Pharmacokinetic Properties of Cthrc1

**Figure 8:**
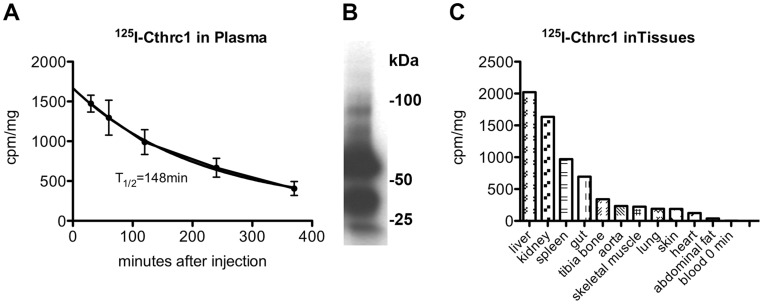
Pharmacokinetic properties of Cthrc1. (A) Clearance curve of ^125^I-Cthrc1 injected intra-arterially into Cthrc1 null mice. The half-life of the protein was calculated to be 146 min. (B) SDS-PAGE and autoradiographic analysis of plasma 30 min after ^125^I-Cthrc1 injection. Monomeric, dimeric and higher molecular weight forms of Cthrc1 were detectable. (C) ^125^I-Cthrc1 content in various tissues 6 h after injection.

Immunoblotting of plasma from Cthrc1 transgenic mice [1] revealed that the pharmacokinetic properties of Chtrc1 are compatible with a role of a circulating factor (Fig. 2A). To determine the half-life of Cthrc1 in circulation, we injected ^125^I-labeled Cthrc1 into the carotid artery of Cthrc1 null mice and obtained the clearance curve shown in Fig. 8A. The half-life was calculated to be 146 minutes. The integrity of the injected radiolabeled Cthrc1 after 30 minutes in circulation was examined by subjecting plasma to SDS-PAGE under non-reducing conditions followed by autoradiography. Apparent monomeric and dimeric forms of the protein were dominant with higher molecular weight oligomers also detectable (Fig. 8B). Six hours after the Cthrc1 injection the amount of Cthrc1 bound in various organs was determined. Per mg tissue the highest levels of bound Cthrc1 were found in the liver (Fig. 8C). Smaller radiolabeled Cthrc1 fragments were detected in the urine suggesting that at least some of the radioactivity detected in the kidney represents degraded, excreted Cthrc1 and not bound Cthrc1 (data not shown).

### Cthrc1 in Human Plasma

**Figure 9:**
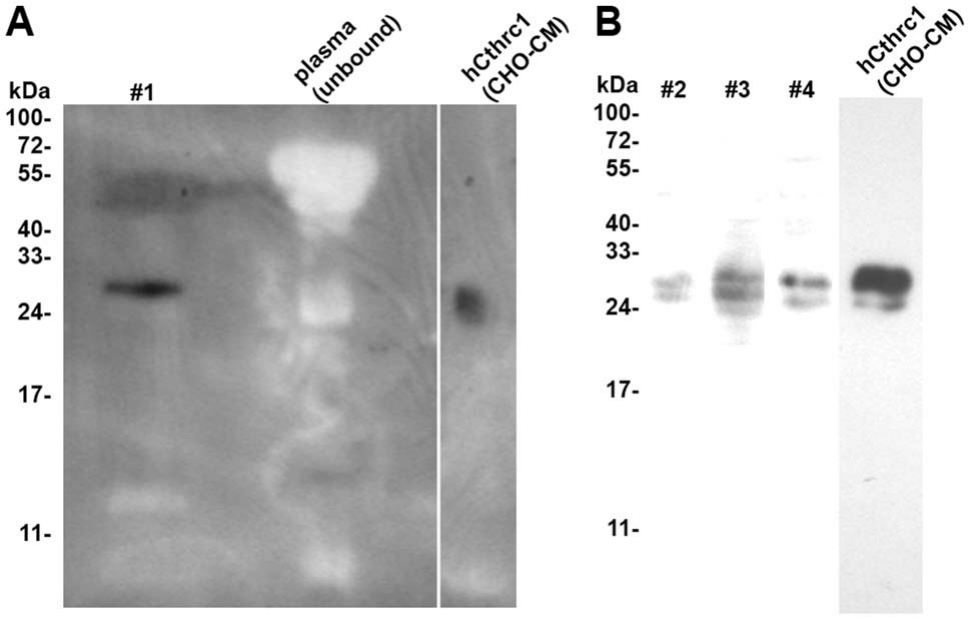
Cthrc1 detection in human plasma. A pull-down assay with monoclonal Cthrc1 antibody coupled to magnetic beads was performed on human plasma samples (#1–#4). (A) The eluate was analyzed by Western blotting with HRP-conjugated anti-Cthrc1 antibody, conditioned medium from hCthrc1 expressing CHO-K1 cells was used as a positive control. No Cthrc1 was detectable in the unbound plasma fraction (note absence of non-specific bands). (B) The eluates from plasma samples #2–#4 were immunoblotted with biotin-conjugated anti-Cthrc1 antibody followed by HRP-conjugated streptavidin.

We generated mouse monoclonal antibodies suitable for detection of Cthrc1 by ELISA. These antibodies conjugated to magnetic beads were used to isolate Cthrc1 from 15 ml of plasma. For one sample (#1) (Fig. 9A) the eluate from the beads was examined for the presence of Cthrc1 by Western blotting with HRP-conjugated anti-Cthrc1 antibodies showing a single band of Cthrc1 with a similar molecular mass as Cthrc1 expressed in CHO-K1 cells (Fig. 9A). Three more plasma samples from different donors (#2, #3, #4) were analyzed in the same manner with the exception that the eluates from the magnetic beads were immunoblotted with biotin-conjugated anti-Cthrc1 followed by HRP-conjugated streptavidin (Fig. 9B). Similar results were obtained as shown in Fig. 9A.

## Discussion

We originally discovered Cthrc1 in a screen for genes upregulated in balloon-injured arteries, where it was highly induced in activated adventitial cells. Database searches and sequence alignments revealed that Cthrc1 is a highly conserved and unique gene in chordates with no homologues found in lower species such as flies or worms [1]. With adiponectin and other members of the C1q/TNF-related protein (CTRP) family Cthrc1 shares the presence of a short N terminal collagen domain but it lacks the characteristic C1q domain that defines the CTRP family of proteins [9]. Cthrc1 and adiponectin are similar in molecular mass, 243 and 244 amino acids, respectively. Like adiponectin, we demonstrate here that Cthrc1 is a circulating hormone that forms high molecular weight complexes [10], [11], although the significance of the different Cthrc1 molecular weight complexes needs to be determined.

**Figure 10:**
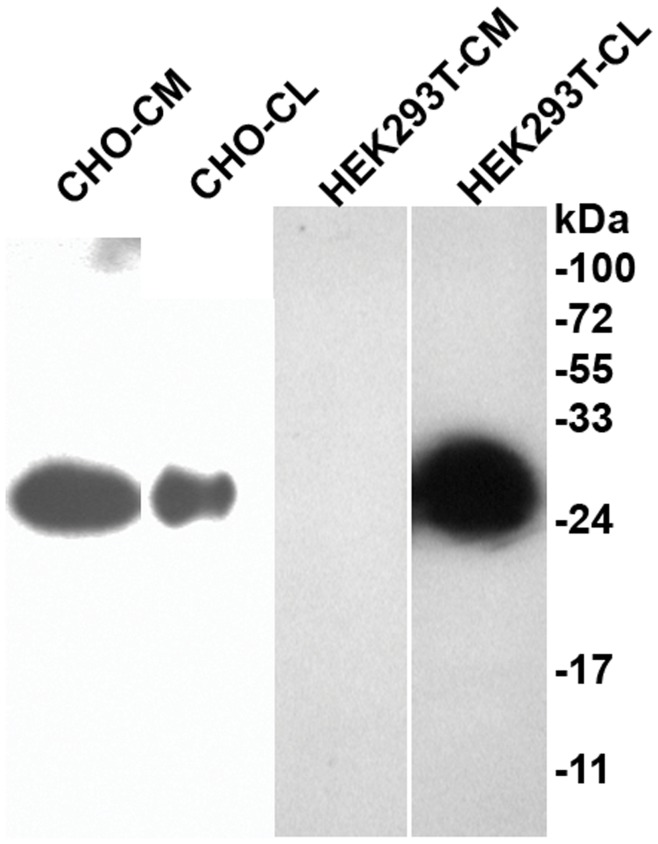
Cthrc1 secretion is cell type dependent. Detection of Cthrc1 in conditioned medium (CM) and cell lysate (CL) of CHO-K1 or HEK293T cells 72 h after transfection with a Cthrc1 expression vector. Note the absence of Cthrc1 in the CM of HEK293T cells.

Yamamoto et al. [2] reported an interaction of Cthrc1 with the planar cell polarity pathway (PCP) of non-canonical WNT signaling. The authors’ reasoning for investigating an involvement of Cthrc1 in the PCP pathway was based on the fact that the Cthrc1 gene is located adjacent to the Frizzled6 gene, which is a WNT binding receptor. The targeted replacement of the first exon of the Cthrc1 gene by a LacZ reporter gene in mice was reported to demonstrate expression of Cthrc1 in inner ear hair cells [2]. Abnormalities in inner ear development occurred when Cthrc1 null mice were crossed with mice carrying one mutant allele of Vangl2, but these abnormalities were only observed when these compound mutants were on a specific mixed genetic background and not when the mutants were crossed with outbred mice [2]. With our antibodies we have been unable to detect expression of Cthrc1 in inner ear hair cells and auditory testing did not reveal any hearing abnormalities in our mutant mice (data not shown). The use of LacZ expression as a surrogate for endogenous Cthrc1 localization has obvious limitations when the endogenous protein is released into the circulation. Yamamoto et al. [2] performed most of their Cthrc1 protein interaction studies in HEK293T cells. Unlike Cthrc1 transfected CHO-K1 cells, which secrete Cthrc1 readily into the medium, Cthrc1 cannot be detected in the medium of transfected HEK293T cells (Fig. 10). This raises the question of suitability of the HEK293T cell line for studying Cthrc1 in vitro. Detection of Cthrc1 in plasma implies that it is secreted from cells. In the neurosecretory nuclei of the hypothalamus (Fig. 6A–C) and the paraventricular cells shown in Fig. 6M, we observed immunoreactivity with granular appearance restricted to the cytoplasm and axon of the cell. This is suggestive of Cthrc1 packaging in secretory granules, a characteristic of a regulated release mechanism that has been described for the release of neuropeptides from neuroendocrine cells [12]. A role for carboxypeptidase E (CPE) in the transport of peptide hormone-containing vesicles to the site of release has been demonstrated in this process [13]. An interaction of Cthrc1 with CPE still needs to be demonstrated. The presence of a C terminal lysine residue in the Cthrc1 sequence would be compatible with an interaction with CPE. We examined the entire midbrain region with the attached pituitary by serial sectioning and Cthrc1 immunohistochemistry both in mice and pigs but we were unable to obtain evidence for transport of Cthrc1 expressed in the hypothalamus along axons to the pituitary gland. CPE also cleaves off C terminal lysine or arginine residues and if this is the case for Cthrc1, it could affect the ability of the C terminal specific antibody (Vli-55) to detect Cthrc1 by immunohistochemistry. Thus, if the C terminal lysine gets cleaved during the transport of the protein along axons to the posterior pituitary we might be unable to detected it with the Vli-55 antibody. Finding Cthrc1 in small vessels near sites of Cthrc1 expression (Fig. 6N) provides indirect evidence for the release of Cthrc1 into the circulation.

Here we generated a novel Cthrc1 null mutant mouse and focused on the characterization of its phenotype in adulthood. Unlike the two other reported targeted Cthrc1 mutants [2], [3] we replaced 3 of the 4 exons with a neomycin cassette and confirmed that Cthrc1 mRNA was not expressed. Using both N terminal and C terminal specific antibodies no Cthrc1 protein was detectable, ruling out the possibility of a hypomorph phenotype. In agreement with the published Cthrc1 null mutants, our Cthrc1 null mice were also viable and showed no obvious developmental abnormalities.

**Figure 11:**
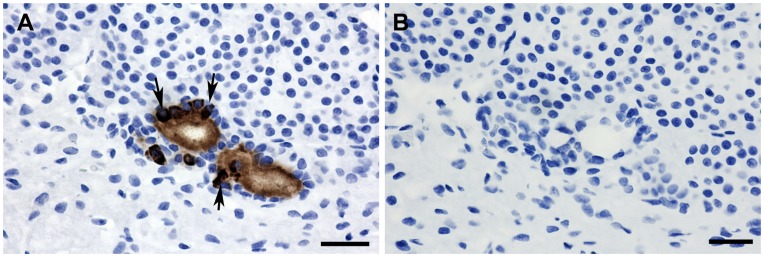
Isolated cells in the rat pituitary express Cthrc1. (A) Cthrc1 immunohistochemistry on pituitary glands from three month old male Sprague Dawley rats identified Cthrc1 expression by isolated cells. Cytoplasmic immunoreactivity is clearly detectable in cells adjacent to extracellular accumulations of Cthrc1 (arrows), suggesting Cthrc1 synthesis by these cells. (B) Pre-absorption of antibody with peptide antigen completely eliminates staining on an adjacent section. Scale bar = 50 µm.

In summary, our study identifies Cthrc1 as a novel circulating hormone with metabolic effects. Expression in the hypothalamus, pituitary gland and remodeling tissues are likely to contribute to Cthrc1 plasma levels.

While Cthrc1 expression was not detectable in the liver and skeletal muscle of the wild type, examination of these organs in our Cthrc1 mutant mice on the C57BL/6J background revealed excessively fatty livers and increased glycogen levels in skeletal muscle and livers of Cthrc1 null mice on the 129S6/SvEv background. This ultimately led us to consider the possibility that Cthrc1 functions as a hormone. We have currently no data related to the mechanism how Cthrc1 affects these organs but our findings do suggest that hepatocytes and myocytes in vivo may express a receptor for Cthrc1 and the binding of ^125^I-Cthrc1 to the liver supports this concept. The Cthrc1 null mutant mice examined here were derived from matings of homozygous null mice. Therefore, we cannot rule out the possibility that some of the metabolic abnormalities seen in the null mutants were due to maternal influences. However, litter sizes of the wild type and the homozygous null matings were comparable and we did not see any evidence of early postnatal failure to thrive on any genotype.

With a sensitive monoclonal anti-Cthrc1 antibody suitable for detection by ELISA we succeeded in demonstrating the presence of Cthrc1 in plasma. Working with plasma we deliberately avoided the use of secondary anti-IgG antibodies because even minimal cross-reactivity with human immunoglobulins could make discrimination between Cthrc1 and the similarly migrating band of the immunoglobulin light chain difficult on a Western blot. As shown in Fig. 2A and Fig. 9, no non-specific bands are detected with HRP-conjugated monoclonal anti-Cthrc1 antibodies in plasma samples by Western blotting. Detection of Cthrc1 in plasma of Cthrc1 transgenic mice and a half-life of approximately 2.5 hours in circulation provide additional support for Cthrc1 as a circulating factor. Our magnetic bead-based pull-down assay was designed to provide a proof of principle and a double antibody sandwich ELISA obviously needs to be developed for a high throughput quantitative screening assay for Cthrc1 in plasma. Our monoclonal antibodies that can detect native Cthrc1 by ELISA do not cross-react with mouse Cthrc1 and in addition, relatively large amounts of plasma were necessary to detect Cthrc1 in human plasma. Therefore, we have not been able to demonstrate the presence of Cthrc1 in mouse plasma. In the absence of a quantitative assay, we can only estimate the Cthrc1 levels detected in the plasma sample. Based on experience with the antibodies and the levels of Cthrc1 expressed by transduced CHO-K1 cells we estimate the levels of Cthrc1 in the plasma sample analyzed here to be below 100 pg/ml, which would be several orders of magnitude lower than those of adiponectin (typically several µg/ml) [14].

The current study also sheds light on the identity of colloid-filled follicles and the anterior pituitary as a source of Cthrc1. In guinea pigs, the first few colloid follicles of the anterior pituitary are detected at the age of 6 months with an average of just over 4 µm in size [6]. They increase in size and number with age and are found in many vertebrates including humans [5], [6]. Focusing on the pig pituitary, here we identify follicles as well as the pituitary cleft separating the anterior lobe from the pars intermedia as storage sites for Cthrc1. However, not all accumulations of Cthrc1 in the pituitary were encapsulated by folliculostellate cells. Staining of adjacent sections with hematoxylin and eosin suggests that Cthrc1 originates from chromophobe cells (Fig. 7), which are thought to represent acidophil and basophilic cells that recently released their secretory vesicles. Our data indicate that chromophobe cells may be the primary source of Cthrc1 in the pituitary. We saw no expression of Cthrc1 in the pituitary of young adult mice and this raises the question whether the pituitary becomes a more significant provider of Cthrc1 with age when tissue remodeling is limited. Alternatively, the origin of Cthrc1 could differ depending on the species and with the pig physiology being more similar to the human physiology, we expect our findings consistently seen in the pig to be more relevant to humans. To further address species-dependent expression of Cthrc1, pituitary glands from three month old male Sprague Dawley rats were examined and isolated foci of Cthrc1 expression by cells surrounding Cthrc1 accumulations were found (Fig. 11). Cthrc1 is expressed in many mesenchyme-derived cells during growth and tissue remodeling especially the skeletal system, where it continues to be expressed in adult bone (Fig. 6O), a tissue that constantly undergoes remodeling. With bone mass accounting for a substantial amount of total body mass, it is likely that osteocytes and osteoblasts contribute substantially to circulating Cthrc1 levels. Interestingly, osteocalcin, another hormone derived from osteoblastic cells has recently been shown to regulate glucose metabolism and fat mass [15].

## References

[1] Pyagay P, Heroult M, Wang Q, Lehnert W, Belden J, et al (2005) Collagen triple helix repeat containing 1, a novel secreted protein in injured and diseased arteries, inhibits collagen expression and promotes cell migration. Circ Res 96: 261–268. 15618538 10.1161/01.RES.0000154262.07264.12

[2] Yamamoto S, Nishimura O, Misaki K, Nishita M, Minami Y, et al (2008) Cthrc1 selectively activates the planar cell polarity pathway of Wnt signaling by stabilizing the Wnt-receptor complex. Dev Cell 15: 23–36. 18606138 10.1016/j.devcel.2008.05.007

[3] Kimura H, Kwan KM, Zhang Z, Deng JM, Darnay BG, et al (2008) Cthrc1 is a positive regulator of osteoblastic bone formation. PLoS ONE 3: e3174. 18779865 10.1371/journal.pone.0003174 PMC2527134

[4] LeClair RJ, Durmus T, Wang Q, Pyagay P, Terzic A, et al (2007) Cthrc1 is a novel inhibitor of transforming growth factor-beta signaling and neointimal lesion formation. Circ Res 100: 826–833. 17322174 10.1161/01.RES.0000260806.99307.72

[5] Ogawa S, Couch EF, Kubo M, Sakai T, Inoue K (1996) Histochemical study of follicles in the senescent porcine pituitary gland. Arch Histol Cytol 59: 467–478. 9037383 10.1679/aohc.59.467

[6] Kameda Y (1991) Occurrence of colloid-containing follicles in the pars distalis of pituitary glands from aging guinea pigs. Cell Tissue Res 263: 115–124. 2009544 10.1007/BF00318406

[7] Rosen CJ, Ackert-Bicknell CL, Adamo ML, Shultz KL, Rubin J, et al (2004) Congenic mice with low serum IGF-I have increased body fat, reduced bone mineral density, and an altered osteoblast differentiation program. Bone 35: 1046–1058. 15542029 10.1016/j.bone.2004.07.008

[8] Ciocca DR, Gonzalez CB (1978) The pituitary cleft of the rat: an electron microscopic study. Tissue Cell 10: 725–733. 746543 10.1016/0040-8166(78)90058-7

[9] Wong GW, Krawczyk SA, Kitidis-Mitrokostas C, Ge G, Spooner E, et al (2009) Identification and characterization of CTRP9, a novel secreted glycoprotein, from adipose tissue that reduces serum glucose in mice and forms heterotrimers with adiponectin. FASEB J 23: 241–258. 18787108 10.1096/fj.08-114991 PMC2626616

[10] Lara-Castro C, Luo N, Wallace P, Klein RL, Garvey WT (2006) Adiponectin multimeric complexes and the metabolic syndrome trait cluster. Diabetes 55: 249–259. 16380500

[11] Hada Y, Yamauchi T, Waki H, Tsuchida A, Hara K, et al (2007) Selective purification and characterization of adiponectin multimer species from human plasma. Biochem Biophys Res Commun 356: 487–493. 17368570 10.1016/j.bbrc.2007.03.004

[12] Park JJ, Koshimizu H, Loh YP (2009) Biogenesis and transport of secretory granules to release site in neuroendocrine cells. J Mol Neurosci 37: 151–159. 18607778 10.1007/s12031-008-9098-y

[13] Park JJ, Cawley NX, Loh YP (2008) Carboxypeptidase E cytoplasmic tail-driven vesicle transport is key for activity-dependent secretion of peptide hormones. Mol Endocrinol 22: 989–1005. 18202146 10.1210/me.2007-0473 PMC2276472

[14] Lihn AS, Pedersen SB, Richelsen B (2005) Adiponectin: action, regulation and association to insulin sensitivity. Obes Rev 6: 13–21. 15655035 10.1111/j.1467-789X.2005.00159.x

[15] Lee NK, Sowa H, Hinoi E, Ferron M, Ahn JD, et al (2007) Endocrine regulation of energy metabolism by the skeleton. Cell 130: 456–469. 17693256 10.1016/j.cell.2007.05.047 PMC2013746

